# Cutaneous Necrosis of the Skin Secondary to Methicillin-Resistant Staphylococcus aureus (MRSA) and Bacteroides With Superimposed Herpes Simplex Virus Type II (HSV-II) in the Setting of Infective Endocarditis

**DOI:** 10.7759/cureus.38857

**Published:** 2023-05-10

**Authors:** Adrian M Alonso, Eric J Basile, Addie Walker, Basil Patel

**Affiliations:** 1 Internal Medicine, University of Florida College of Medicine, Gainesville, USA; 2 Pathology, University of Florida College of Medicine, Gainesville, USA

**Keywords:** systemic lupus erythematosus, antiphospholipid syndrome, sle, hsv ii, bacteroides, mrsa, cutaneous necrosis, endocarditis

## Abstract

Herpes simplex virus type II (HSV-II) with superimposed bacterial skin infection is an uncommon presentation of cutaneous necrosis in the setting of infective endocarditis. This case reflects a unique presentation of an immunosuppressed patient with infective endocarditis complicated by septic emboli and cutaneous skin lesions attributable to HSV-II and superimposed bacterial skin infection. The patient presented from an outside hospital with symptoms consistent with acute onset heart failure and skin lesions. Transthoracic and transesophageal echocardiography performed there demonstrated focal thickening of the anterior mitral valve leaflet with severe mitral regurgitation. The patient then underwent extensive infectious work-up and was put on broad-spectrum antibiotics. Further work-up demonstrated greater than three DUKE minor criteria and reiterated the focal thickening of the anterior leaflet of the mitral valve, making infective endocarditis the most likely etiology. Biopsies of the skin lesions were performed which stained positive for HSV-II and grew methicillin-resistant Staphylococcus aureus and Bacteroides fragilis. The cardiothoracic surgery service ultimately decided not to perform any surgical intervention to the mitral valve during her hospitalization as she was deemed to be too high of a risk due to her thrombocytopenia and significant comorbidities. She was later discharged in hemodynamically stable condition on long-term intravenous antibiotics with repeat echocardiography demonstrating significant reduction in the mitral regurgitation and the focal thickening of the anterior leaflet of the mitral valve.

## Introduction

Infective endocarditis (IE) is a rare but serious condition with an estimated incidence of 3-10 cases per 100,000 people per year in the United States. It typically occurs in patients with underlying heart disease or prosthetic heart valves and is associated with an elevated risk of morbidity and mortality [1]. Cutaneous manifestations vary, occurring in up to 25% of cases, and can range from small petechiae and purpura to large, painful nodules and ulcers. 

The cutaneous manifestations typically associated with IE are Janeway lesions and Osler nodes - nontender macules or papules on the soles and palms of the feet and painful immunogenic skin lesions, respectively. Other manifestations of IE include splinter hemorrhages, which often manifest as painless erythematous lesions in the nailbed [2]. Rarely, cutaneous necrosis can form as complications from septic emboli from the underlying IE [3]. The underlying histology of these lesions tend to be micro-emboli with abscess formation that may eventually grow the underlying organism when cultured [4]. Common organisms that cause IE-associated cutaneous skin infections include aerobic and anaerobic organisms, namely Staphylococcus aureus and Bacteroides fragilis [5,6]. Mixed cutaneous necrotic infections, such as those with superimposed herpes simplex virus type II (HSV-II) infection in the setting of an immunosuppressed patient with IE, can further complicate the disease course. Understanding the underlying pathophysiology, risk factors, and clinical presentation of cutaneous necrosis associated with methicillin-resistant Staphylococcus aureus (MRSA) and Bacteroides with superimposed HSV-II is crucial for timely diagnosis and management of this rare, potentially life-threatening condition. 

## Case presentation

A 47-year-old Caucasian female with a past medical history of systemic lupus erythematosus (SLE) and antiphospholipid syndrome (APS) on chronic anticoagulation therapy presented to an outside hospital for shortness of breath and significant dyspnea on exertion. A transesophageal echocardiogram (TEE) there demonstrated findings consistent with severe mitral valve regurgitation and focal thickening of the anterior leaflet of the mitral valve. Given her autoimmune history and initial negative cultures for bloodstream infection, there was some suspicion for Libman-Sacks endocarditis. There was additional concern for an active SLE flare and catastrophic antiphospholipid syndrome (CAPS); therefore, the patient was treated with steroids, intravenous immunoglobulins (IVIG), and plasmapheresis. On transfer, she was evaluated by the cardiothoracic surgery service, who postponed immediate mitral valve surgical replacement given her severe thrombocytopenia with overall pancytopenia. Her hospital course was complicated by a variety of infectious processes, including bacteremia caused by Klebsiella oxytoca, Enterococcus faecium, Klebsiella pneumonia, Cytomegalovirus (CMV), Epstein-Barr virus (EBV), as well as possible CMV colitis. She was placed on cefepime, daptomycin, and intravenous ganciclovir. The patient began to develop cutaneous lesions consistent with splinter hemorrhages and Janeway lesions. Repeat blood cultures were negative - though this was in the setting of multiple days on broad-spectrum antibiotics. A repeat TEE was ordered which re-demonstrated the mitral valve thickening previously seen from the outside hospital's TEE along with mildly dilated left atrium, and a left ventricular ejection fraction of 55%. As the patient had persistent fevers, vascular phenomenon, previously positive blood cultures from other organisms, and echocardiographic abnormalities, there was now increasing concern for infectious endocarditis since that fulfills three or more of the DUKE minor criteria for infective endocarditis.

CT-angiogram of the aorta with intravenous contrast demonstrated new tiny focal hypodensity in the left common iliac artery, possibly representing thrombus/embolus. There was also, of note, a focal wedge-shaped hypodensity of the posterior spleen suspicious for developing infarct. CT of the chest demonstrated new bilateral soft tissue pulmonary nodules, greater in the lower lobes, that were suspicious for septic emboli vs. metastatic disease. The cardiothoracic surgery service was re-consulted, given these new findings, and ultimately postponed surgical intervention to the mitral valve citing her significant comorbidities, thrombocytopenia, and her being too high risk for intra- and post-operative complications.

**Figure 1:**
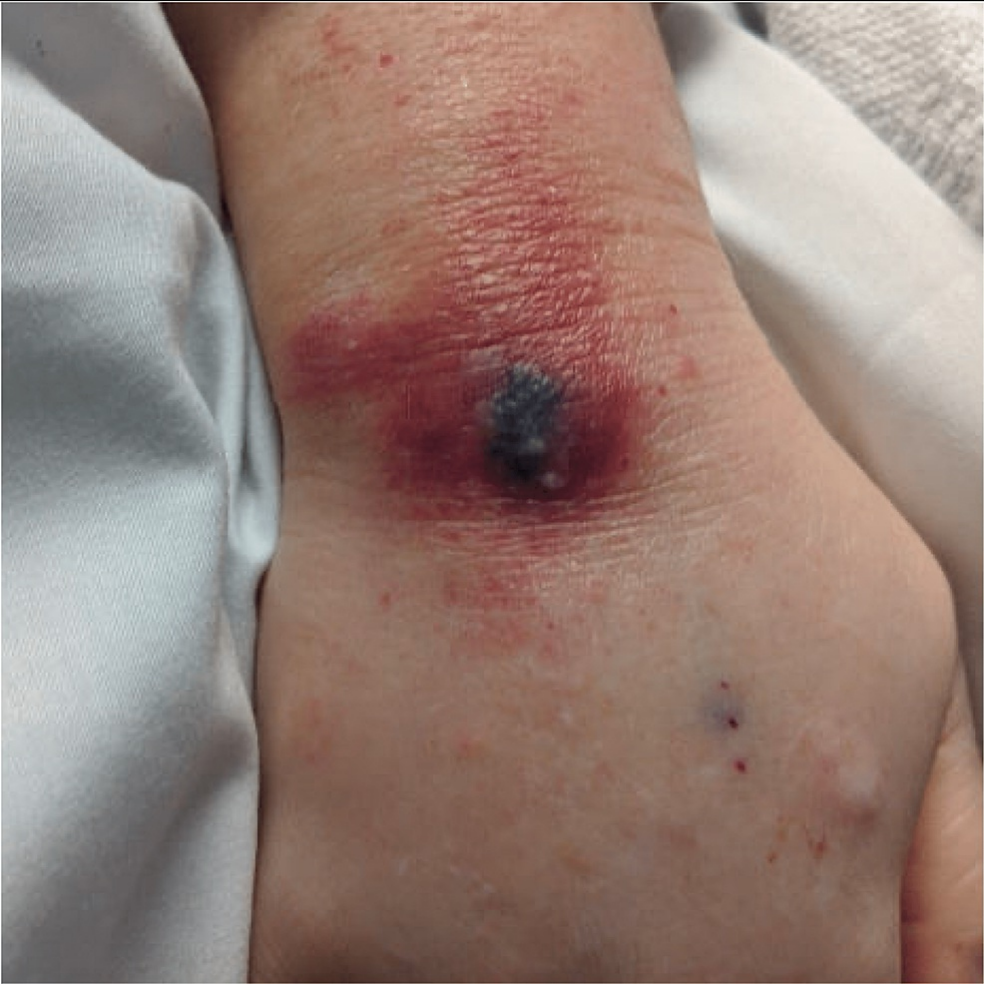
Cutaneous, necrotic region of right wrist where punch biopsy was performed.

**Figure 2:**
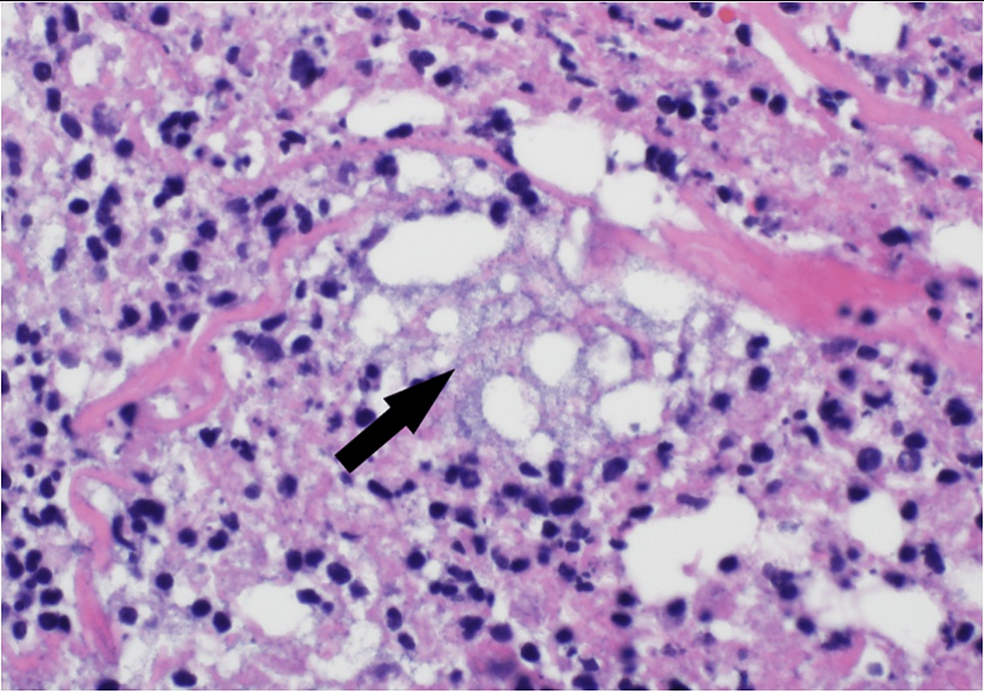
Histology of patient's skin biopsy demonstrating multinucleated cell exhibiting nuclear molding and margination of chromatin (black arrow) consistent with herpesvirus cytopathic effect.

**Figure 3:**
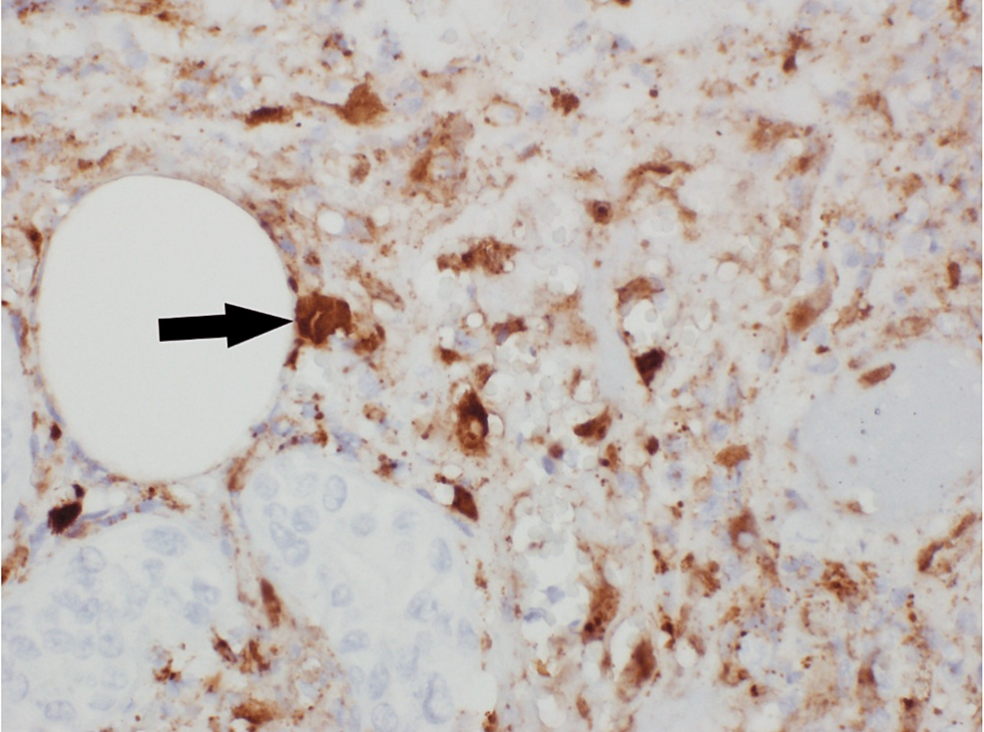
Immunohistochemical staining for herpes simplex virus I/II demonstrating nuclear positivity within a multinucleated cell (black arrow).

**Figure 4:**
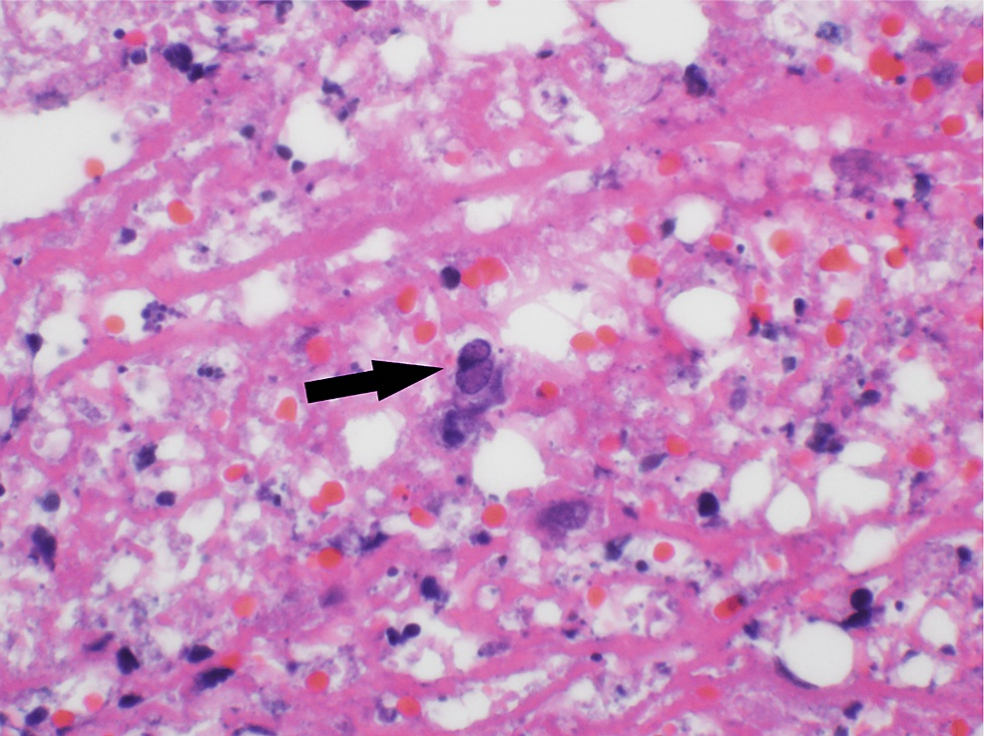
Histology of patient's skin biopsy demonstrating multinucleated cell exhibiting nuclear molding and margination of chromatin (black arrow) consistent with herpesvirus cytopathic effect.

An erythematous lesion was noted on the dorsal aspect of her right wrist on hospital day 8 (Figure 1). There was initial concern that this was secondary to her arterial line, which was recently removed, or cutaneous necrosis secondary to her underlying IE. The dermatology service performed a punch biopsy of this lesion and histopathology demonstrated numerous gram-positive bacteria in the deep dermis on Grocott methenamine silver (GMS) and periodic acid-Schiff (PAS) stain. Additionally, there was deep abscess formation, necrosis, and hemorrhage present. There were also scattered multinucleated cells with clear nuclear molding and margination of chromatin was identified in the deep dermis, both within the inflammatory infiltrate and within the necrotic vessel walls. These findings were consistent with a herpesvirus infection with superimposed bacterial infection. Immunohistochemistry confirmed HSV-II infection, and bacterial cultures were positive for Bacteroides fragilis and MRSA (Figures 2, 3, 4). The patient was already being treated with broad-spectrum antimicrobials that covered these organisms. Due to the patient's status of being a poor surgical candidate, she was discharged on long-term intravenous antibiotics in hemodynamically stable condition after a prolonged and complicated hospital course. A repeat outpatient TEE one month later demonstrated significant interval resolution of the mitral valve thickening and the patient had significant clinical improvement.

## Discussion

This case demonstrates a unique presentation of cutaneous skin necrosis caused by polymicrobial infection, including HSV-II. Bacteroides and MRSA are well-established organisms that can cause necrotizing fasciitis with abscess formation [7]. However, as blood cultures did not demonstrate these species, there is no objective measure of confirmation that these organisms migrated from the presumed seeded valve to the skin. The likely primary site of infectious origin was believed to be from a previous arterial line or peripheral catheter. However, given the patient's systemic septic emboli to her lungs, brain, spleen, and skin findings of Janeway lesions and splinter hemorrhages, cutaneous septic emboli were unable to be definitively ruled out. 

HSV-II is commonly associated as the leading causative agent for anogenital lesions and is known to reactivate in times of physiological stress and immunosuppression. Cutaneous manifestations outside of the anogenital region usually manifest as herpetic whitlow vesicles [8]. Although rare, HSV-II can cause cutaneous necrosis, as evidenced by a previous case reporting HSV-II nasal reactivation causing necrosis with a superimposed bacterial infection [9]. Other herpes virus families, such as herpes zoster virus (HZV), have also been shown to cause necrotizing fasciitis when superimposed with a bacterial infection [10]. This current case further demonstrates the complexity and clinical challenge of HSV-II regional cutaneous necrosis as bacteria also grew, clouding the picture of a causative organism. This is yet another unique presentation of a cutaneous lesion in the setting of IE, immunosuppression, and acute illness. 

This cause further demonstrates the need for timely diagnosis of causative organisms in cutaneous necrosis to treat appropriately. There is also further elucidation of HSV-II, causing yet another cutaneous manifestation of the virus. The medical team acted quickly by performing a punch biopsy that revealed underlying HSV-II and an anaerobic infection. 

## Conclusions

This case demonstrates a rare multi-factorial complication of infective endocarditis in a patient with immunosuppression and subsequent HSV-II reactivation. Further, it demonstrates a range in skin infection manifestations that can be used to increase suspicion for multiple contributing organisms in this population subgroup. Previous literature seems lacking in describing and attributing causal organisms to this type of lesion. 

This patient population, as well as their providing physicians, would benefit from more research and classification of these types of lesions as this would promote increased expediency in appropriate antibiotic use. 
